# A Visualized Dynamic Prediction Model for Overall Survival in Elderly Patients With Pancreatic Cancer for Smart Medical Services

**DOI:** 10.3389/fpubh.2022.885624

**Published:** 2022-05-24

**Authors:** Jiang Zhong, XingShu Liao, Shuang Peng, Junyi Cao, Yue Liu, Chunyang Liu, Ju Qiu, Xiaoyan Guan, Yang Zhang, Xiaozhu Liu, Shengxian Peng

**Affiliations:** ^1^College of Computer Science, Chongqing University, Chongqing, China; ^2^General Affairs Section, The People's Hospital of Tongnan District, Chongqing, China; ^3^Department of Medical Quality Control, First People's Hospital of Zigong City, Zigong, China; ^4^Department of Pediatrics, First People's Hospital of Zigong City, Zigong, China; ^5^Scientific Research Department, First People's Hospital of Zigong City, Zigong, China; ^6^College of Medical Information, Chongqing Medical University, Chongqing, China; ^7^Department of Cardiology, The Second Affiliated Hospital of Chongqing Medical University, Chongqing, China

**Keywords:** nomogram, elderly patients, pancreatic cancer, SEER database, online application

## Abstract

**Background:**

Pancreatic cancer (PC) is a highly malignant tumor of the digestive system. The number of elderly patients with PC is increasing, and older age is related to a worse prognosis. Accurate prognostication is crucial in treatment decisions made for people diagnosed with PC. However, an accurate predictive model for the prognosis of these patients is still lacking. We aimed to construct nomograms for predicting the overall survival (OS) of elderly patients with PC.

**Methods:**

Patients with PC, older than 65 years old from 2010 to 2015 in the Surveillance, Epidemiology, and End Results database, were selected and randomly divided into training cohort (*n* = 4,586) and validation cohort (*n* = 1,966). Data of patients in 2016–2018 (*n* = 1,761) were used for external validation. Univariable and forward stepwise multivariable Cox analysis was used to determine the independent prognostic factors. We used significant variables in the training set to construct nomograms predicting prognosis. The performance of the models was evaluated for their discrimination and calibration power based on the concordance index (C-index), calibration curve, and the decision curve analysis (DCA).

**Results:**

Age, insurance, grade, surgery, radiation, chemotherapy, T, N, and American Joint Commission on Cancer were independent predictors for OS and thus were included in our nomogram. In the training cohort and validation cohort, the C-indices of our nomogram were 0.725 (95%CI: 0.715–0.735) and 0.711 (95%CI: 0.695–0.727), respectively. The 1-, 3-, and 5-year areas under receiver operating characteristic curves showed similar results. The calibration curves showed a high consensus between observations and predictions. In the external validation cohort, C-index (0.797, 95%CI: 0.778–0.816) and calibration curves also revealed high consistency between observations and predictions. The nomogram-related DCA curves showed better clinical utility compared to tumor-node-metastasis staging. In addition, we have developed an online prediction tool for OS.

**Conclusions:**

A web-based prediction model for OS in elderly patients with PC was constructed and validated, which may be useful for prognostic assessment, treatment strategy selection, and follow-up management of these patients.

## Introduction

Pancreatic cancer (PC) is one of the most dangerous malignancies in the world and is a lethal disease with dismal survival rates, with a 5-year overall survival rate of <5% and a median survival of ~6–8 months after diagnosis. It is the fourth most common cause of cancer death in both men and women due to the difficulty of early detection and poor prognosis (1–3). In addition, PC is the main disease of the elderly population, as aging intensifies, the number of elderly with PC is expected to rise in the future. At the same time, it is difficult for physicians to stage and predict the prognosis of PC because it exhibits a variety of tumor behaviors and symptoms (4). Despite the continuous introduction of new surgical techniques and medical therapies, and the evolving concept of integrated management of PC, the prognosis of PC patients has improved modestly, but the 5-year survival rate has not improved significantly (5). So the poor prognosis of PC remains a major challenge for mankind.

The tumor-node-metastasis (TNM) staging system of the American Joint Commission on Cancer (AJCC) 8th edition, is commonly used for prognostic evaluation of pancreatic ductal adenocarcinoma. However, researchers have found the staging system inadequate (6–8). It only incorporates some features of the tumors into the staging system, and its prognostic factors go far beyond these. Shi et al. constructed a nomogram for predicting the overall survival (OS) and cancer-specific survival (CSS) of young patients with PC and found that primary site, pathological types, AJCC stage, and surgery were independent factors affecting CSS (8). Although many studies have confirmed that the clinical characteristics and prognosis of older patients with PC are different from young patients (9–11), the nomogram of PC in previous studies cannot accurately predict the OS of elderly patients with PC (12–14). Therefore, technically feasible and easily clinically accessible nomograms that predict the OS specifically for elderly patients with PC are still urgently required.

At present, nomograms have been developed and proposed as an innovative alternative tool for prognostic assessment of many cancers (15–17), which can combine important demographic and clinicopathological features to estimate individual survival for patients with cancer. A nomogram for patients with PC over 65 years old or older derived from population-based data, to the best of our knowledge, has never been reported. The Surveillance, Epidemiology, and End Results (SEER) database serves as the definitive cancer database in the United States. It contains data related to cancer prognosis such as age, race, marital status, histologic grade, tumor size, surgery, radiation, and chemotherapy (18). Based on the SEER database, we aim to investigate the risk factors for OS in elderly patients with PC and establish a web-based prediction model for predicting OS, which might be helpful for the prognostic prediction, and treatment strategy selection. It also can help doctors and patients to make follow-up decisions.

## Patients and Methods

### Patients and Variables Inclusion

**Figure 1:**
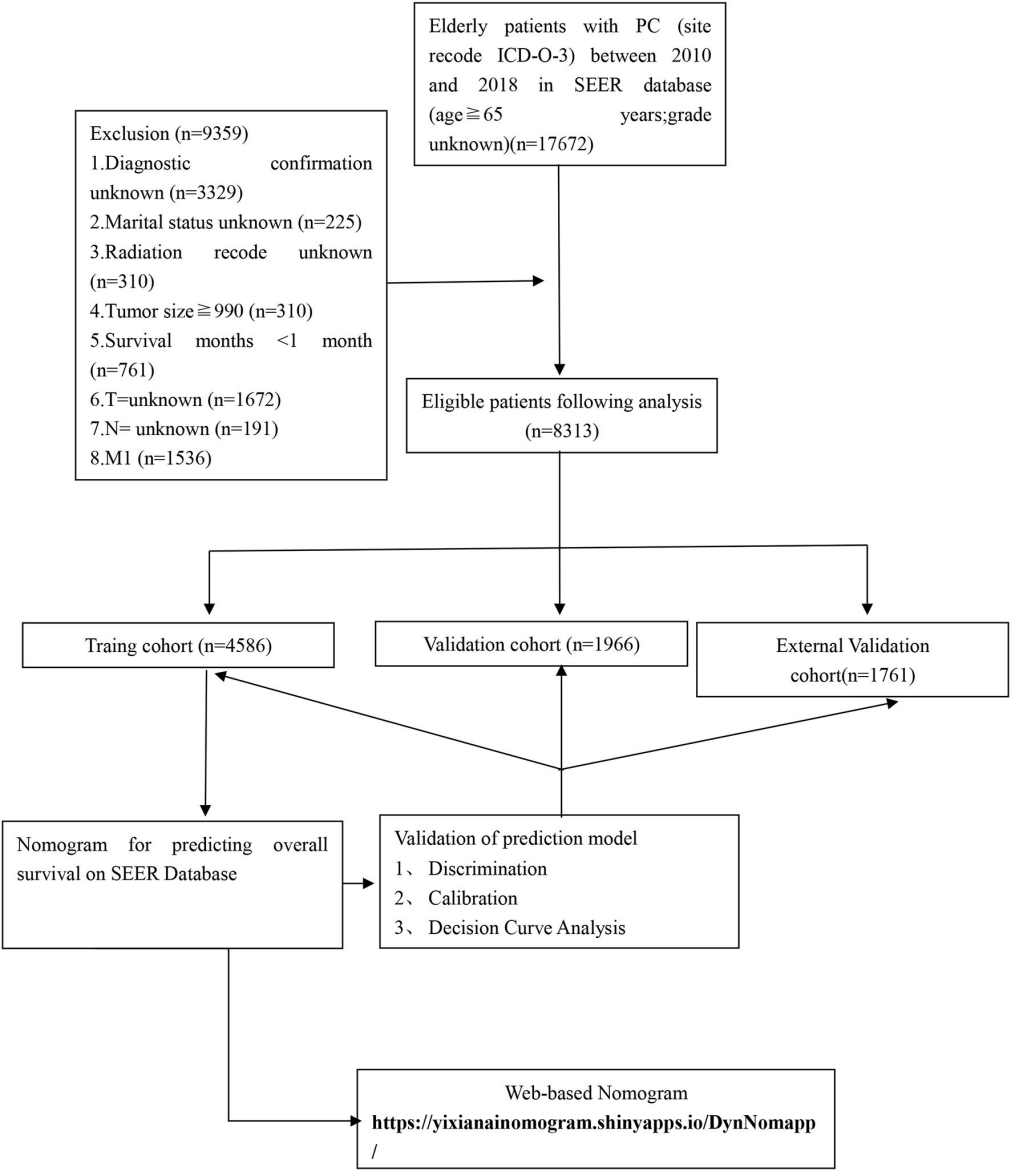
The flowchart of including and dividing patients. Github: https://github.com/xiaoyang11223/pancreatic-cancer.git.

The clinicopathological data of all patients with PC from 2010 to 2018 were downloaded from the SEER database. We put the original data in Supplementary Material and put the relevant code on GitHub, see the link for details https://github.com/xiaoyang11223/pancreatic-cancer.git. It collects information on patient demographics, year of diagnosis, marital status, tumor size, histopathological grade, TNM stage, surgery, radiotherapy, chemotherapy, post-operative AJCC 7th staging, and follow-up for survival. The inclusion criteria were as follows: (1) age≥65years; (2) International Classification of Diseases for Oncology, 3rd edition [ICD-O-3] code 8150, 8151, 8152, 8154, 8155; (3) those with a confirmed pathological diagnosis from 2010 to 2018; and (4) those with unknown data about the grade. The exclusion criteria were as follows: (1) unknown diagnostic confirmation; (2) unknown marital status; (3) unknown insurance recode; (4) survival time <1 month; (5) tumor size≥990; and (6) unknown whether radiotherapy was performed. Finally, 6,552 eligible elderly patients with PC were included in this study. The following variables were analyzed: age, insurance, grade, surgery, radiation, chemotherapy, TNM stage, and AJCC. The detailed flow chart was shown in Figure 1. For this study, we have signed authorization and received permission from SEER to access and use the SEER information. The SEER database is publicly available and the data for all patients are deidentified, so institutional review board approval and informed consent were not required in this study.

### Statistical Analysis

To construct and validate the nomogram and ensure the robustness of the prediction model, we divided the data into the training set and verification set according to 7:3 through the SAMPLE function of R. we randomly assigned 70% (*n* = 4,586) of patients from 2010 to 2015 and 30% (*n* = 1,966) of patients from 2010 to 2015 to the training and validation cohorts, respectively. A total of 1,761 patients in the SEER database from 2016 to 2018 were included in the external validation cohort. A chi-square test was used to compare the clinicopathological characteristics between the training set and the validation set. OS referred to the duration of PC from diagnosis to death, and patients who were alive at the point of the last follow-up were considered censored events. Variables related to OS for which *p* < 0.05 in the univariable analysis or with important clinical value were entered into a multivariate logistic regression model based on proportional subdistribution hazard models to determine risk factors independently associated with the development of PC in elderly patients, which was performed using a Cox proportional hazards regression model. Discrimination of the nomograms was measured by the concordance index (C-index) and receiver operating characteristic (ROC) curve with its respective 95% CI, which quantifies the level of consistency between the observed OS and the predicted OS probability.

Clinical prediction models can provide doctors and patients with quantified risk value based on current health status to predict future health status utilizing non-invasive, low-cost, and easily collected indicators, which has important health economics significance. Decision curve analysis (DCA) is a new calculation method that estimates the net benefits under various risk thresholds to evaluate the clinical value of the model. We used DCA to evaluate the clinical practicability of our nomogram. Based on the cut-off value calculated from the total score of our nomogram, we divided patients into a low-risk group and a high-risk group. We applied Kaplan–Meier curves and log-rank tests to compare patient survival between different groups. The nomogram, C-indices, ROCs, calibration curves, DCA curves, Kaplan–Meier curves, and a web application for survival prediction were by utilizing R software. For all statistical analyses, *p*-value < 0.05 was considered statistically significant.

## Results

### Patients Characteristic

Our study flowchart is shown in Figure 1. Finally, there were a total of 6,552 cases of elderly patients with PC diagnosed in the SEER database between 1 January 2010 and 31 December 2015. These patients were randomly divided into a training set (*N* =4,586) and a validation set (*N* = 1,966). The demographic and clinicopathological information of patients were listed in Table 1. Of these patients, 3,702 cases (56.50%) were 65–74 years old, 2,850 cases (43.50%) were >74 years old, 5,501 (83.96%) were white, 3,284 (50.12%) were male, 5,945 (90.74%) were married, and 4,683 (71.47%) were T3 stage. The histological tumor grades were 1,395 (21.29%), 22,839 (43.33%), 2,209 (33.71%), and 109 (1.66%) for patients with tumor histological grades I, II, III, and IV, respectively. Their AJCC stages were IA: *n*=640 (9.77%), IB: *n*=823 (12.56%), IIA: *n*=1,724 (26.31%), and IIB: *n*=3365 (51.36%), respectively. There were 3,348 (51.10%) underwent local or pancreatectomy, 3,649 (55.69%) underwent chemotherapy, and 5,043 (76.97%) did not receive radiotherapy. Detailed patient clinical characteristics were summarized in Table 1. There were no significant differences in clinicopathologic characteristics between the training and the validation set. Characteristics of patients in the external validation cohort are shown in Supplementary Table S1.

**Table 1:** Demographical and clinical characteristics of training set and validation set.

**Characteristics**	**All patients**	**Training cohort**	**Validation cohort**	***P***
	***N*** **= 6,552**	***N*** **= 4,586**	***N*** **= 1,966**	
Age (%)				0.2609
65–74	3,702 (56.50)	2,570 (56.04)	1,132 (57.58)	
>74	2,850 (43.50)	2,016 (43.96)	834 (42.42)	
Sex (%)				0.48
Female	3,268 (49.88)	2,301 (50.17)	967 (49.19)	
Male	3,284 (50.12)	2,285 (49.83)	999 (50.81)	
Race (%)				<0.001
Black	503 (7.68)	283 (6.17)	220 (11.19)	
Other^a^	548 (8.36)	495 (10.79)	53 (2.70)	
White	5,501 (83.96)	3,808 (83.04)	1,693 (86.11)	
Marital state (%)				0.24
Married	5,945 (90.74)	4,148 (90.45)	1,797 (91.40)	
No	607 (9.26)	438 (9.55)	169 (8.60)	
Insurance (%)				0.0003
Insured	6,056 (92.43)	4,203 (91.65)	1,853 (94.25)	
No	496 (7.57)	383 (8.35)	113 (5.75)	
Grade (%)				0.0834
I	1,395 (21.29)	975 (21.26)	420 (21.36)	
II	2,839 (43.33)	2,016 (43.96)	823 (41.86)	
III	2,209 (33.71)	1,511 (32.95)	698 (35.50)	
IV	109 (1.66)	84 (1.83)	25 (1.27)	
T (%)				0.0025
T1	709 (10.82)	516 (11.25)	193 (9.82)	
T2	1,160 (17.70)	850 (18.53)	310 (15.77)	
T3	4,683 (71.47)	3,220 (70.21)	1,463 (74.42)	
*N* (%)				0.0535
N0	3,187 (48.64)	2,267 (49.43)	920 (46.80)	
N1	3,365 (51.36)	2,319 (50.57)	1,046 (53.20)	
AJCC (%)				0.0833
IA	640 (9.77)	459 (10.01)	181 (9.21)	
IB	823 (12.56)	602 (13.13)	221 (11.24)	
IIA	1,724 (26.31)	1,206 (26.30)	518 (26.35)	
IIB	3,365 (51.36)	2,319 (50.57)	1,046 (53.20)	
Surgery (%)				<0.001
Extended pancreatoduodenectomy	219 (3.34)	122 (2.66)	97 (4.93)	
Local excision of tumor	1,121 (17.11)	777 (16.94)	344 (17.50)	
Local or pancreatectomy	3,348 (51.10)	2,357 (51.40)	991 (50.41)	
No	1,264 (19.29)	936 (20.41)	328 (16.68)	
Total pancreatectomy	600 (9.16)	394 (8.59)	206 (10.48)	
Radiation (%)				0.0001
No	5,043 (76.97)	3,591 (78.30)	1,452 (73.86)	
Yes	1,509 (23.03)	995 (21.70)	514 (26.14)	
Chemotherapy (%)				0.8526
No	2,903 (44.31)	2,028 (44.22)	875 (44.51)	
Yes	3,649 (55.69)	2,558 (55.78)	1,091 (55.49)	
Tumor size, mm [median (IQR)]		31.000 [24.000, 41.000]	31.000 [24.000, 41.000]	0.7675

a *Other includes American Indian/AK Native, Asian/Pacific Islander*.

### Univariable and Multivariable Cox Regression Analysis

Univariate Cox regression analysis was used to screen the prognostic factors of PCC in elderly patients, namely, sex, age, insurance, Fuhrman grade, surgery, radiation, chemotherapy, T stage, N stage, and AJCC stage (Table 2). Then we developed multivariate Cox models using the selected factors to identify independent risk factors. The hazard ratio (HR) is used to quantify its effect on OS. Multivariate analysis found that age, insurance, grade, T stage, N stage, surgery, radiation, chemotherapy, and AJCC stage were significant independent risk factors for the prognosis of patients. The results of univariate and multivariate factors are shown in Table 2.

**Table 2:** Univariable and multivariable Cox regression analysis of OS in training set.

**Variable**	**Univariate analysis**		**Multivariate analysis**	
	**HR (95% CI)**	***P***	**HR (95% CI)**	***P***
Sex				
Female	Reference		Reference	
Male	0.94 (0.88–0.99)	0.029	1.01 (0.96–1.08)	0.662
Race				
Black	Reference		Reference	
Other	0.88 (0.76–1.02)	0.095	0.9 (0.78–1.05)	0.171
White	0.91 (0.81–1.01)	0.08	0.95 (0.85–1.06)	0.319
Age				
>74	Reference		Reference	
65–74	0.70 (0.66–0.74)	<0.001	0.81 (0.76–0.86)	<0.001
Insurance				
Insured	Reference		Reference	
No	1.24 (1.12–1.39)	<0.001	1.13 (1.01–1.26)	0.030
Marital.state				
Married	Reference		Reference	
No	1.09 (0.99–1.21)	0.08	1.07 (0.97–1.19)	0.196
Grade				
I	Reference		Reference	
II	2.16 (1.97–2.37)	<0.001	2.1 (1.9–2.31)	<0.001
III	3.14 (2.86–3.45)	<0.001	2.9 (2.62–3.21)	<0.001
IV	2.85 (2.26–3.59)	<0.001	2.48 (1.96–3.14)	<0.001
Surgery				
Extended pancreatoduodenectomy	Reference		Reference	
Local excision of tumor	0.61 (0.51–0.73)	<0.001	0.94 (0.78–1.13)	0.485
Local or pancreatectomy	1.01 (0.85–1.19)	0.912	1.12 (0.95–1.33)	0.178
No	2.60 (2.19–3.09)	<0.001	3.88 (3.25–4.63)	<0.001
Total pancreatectomy	0.99 (0.82–1.19)	0.889	1.13 (0.93–1.36)	0.210
Radiation				
No	Reference		Reference	
Yes	0.91 (0.85–0.98)	0.01	0.86 (0.8–0.93)	<0.001
Chemotherapy				
No	Reference		Reference	
Yes	0.94 (0.89–1)	0.056	0.64 (0.59–0.68)	<0.001
T				
T1	Reference		Reference	
T2	2.73 (2.35–3.16)	<0.001	1.49 (1.05–2.12)	0.025
T3	3.47 (3.04–3.96)	<0.001	1.89 (1.36–2.63)	<0.001
N				
N0	Reference		Reference	
N1	1.40 (1.32–1.49)	<0.001	2.07 (1.45–2.96)	<0.001
AJCC				
IA	Reference		Reference	
IB	2.72 (2.31–3.21)	<0.001	1.33 (0.91–1.97)	0.145
IIA	3.52 (3.03–4.09)	<0.001	1.51 (1.05–2.17)	0.026
IIB	3.71 (3.21–4.29)	<0.001	0.73 (0.68–0.79)	<0.001

### Nomogram Construction for 1-, 3-, and 5-Year OS

**Figure 2:**
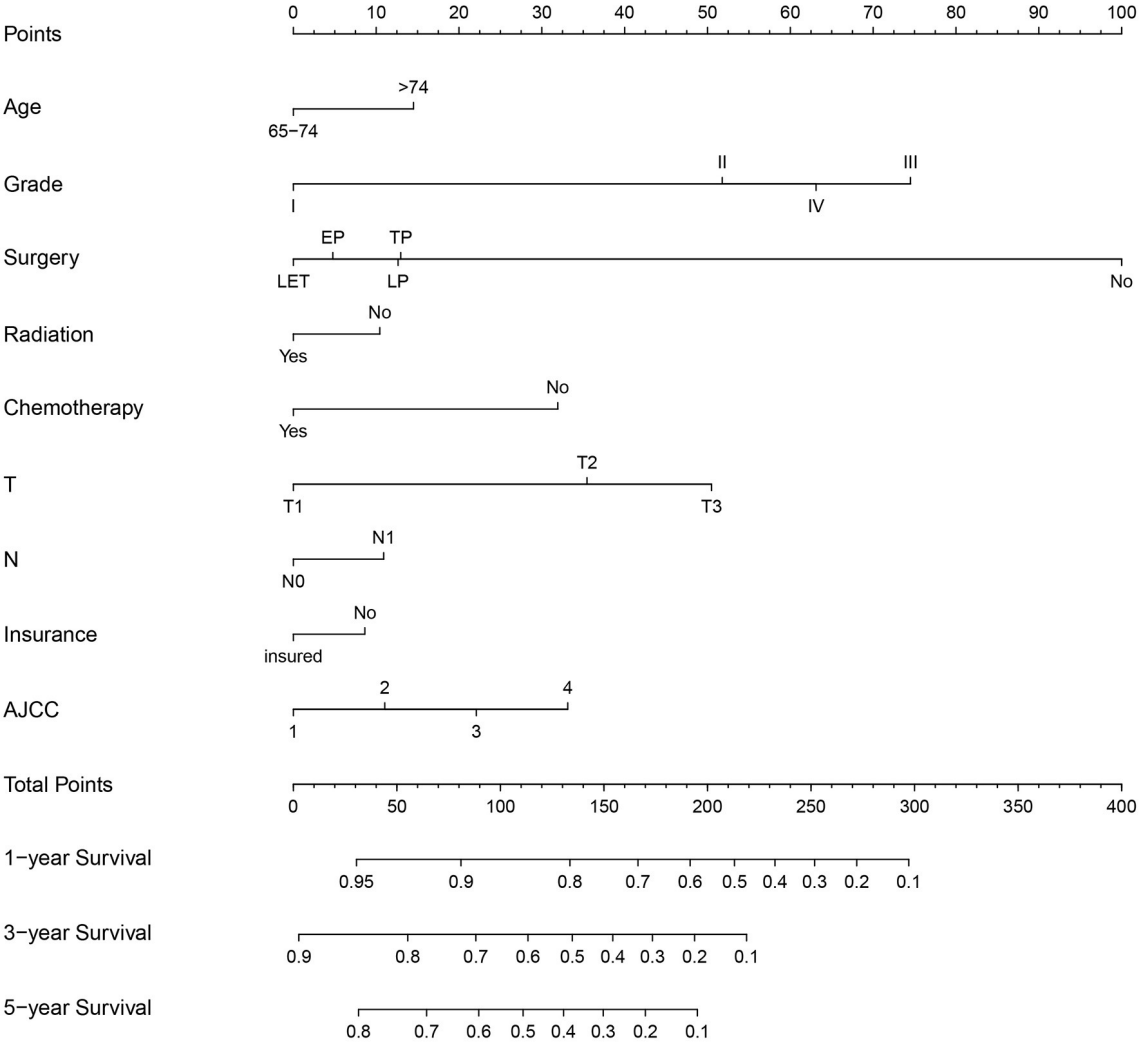
Nomogram for 1-, 3-, and 5-year OS of elderly patients with PC.

The multivariate Cox regression analysis identified the independent risk factors used to construct a nomogram to predict elderly patients with PC at 1-, 3-, and 5-year OS (Figure 2). The nomogram indicated that surgery and grade are still the most significant factors affecting the prognosis of patients, followed by age, T stage, AJCC stage, and chemotherapy.

### Validation of the Nomogram

**Figure 3:**
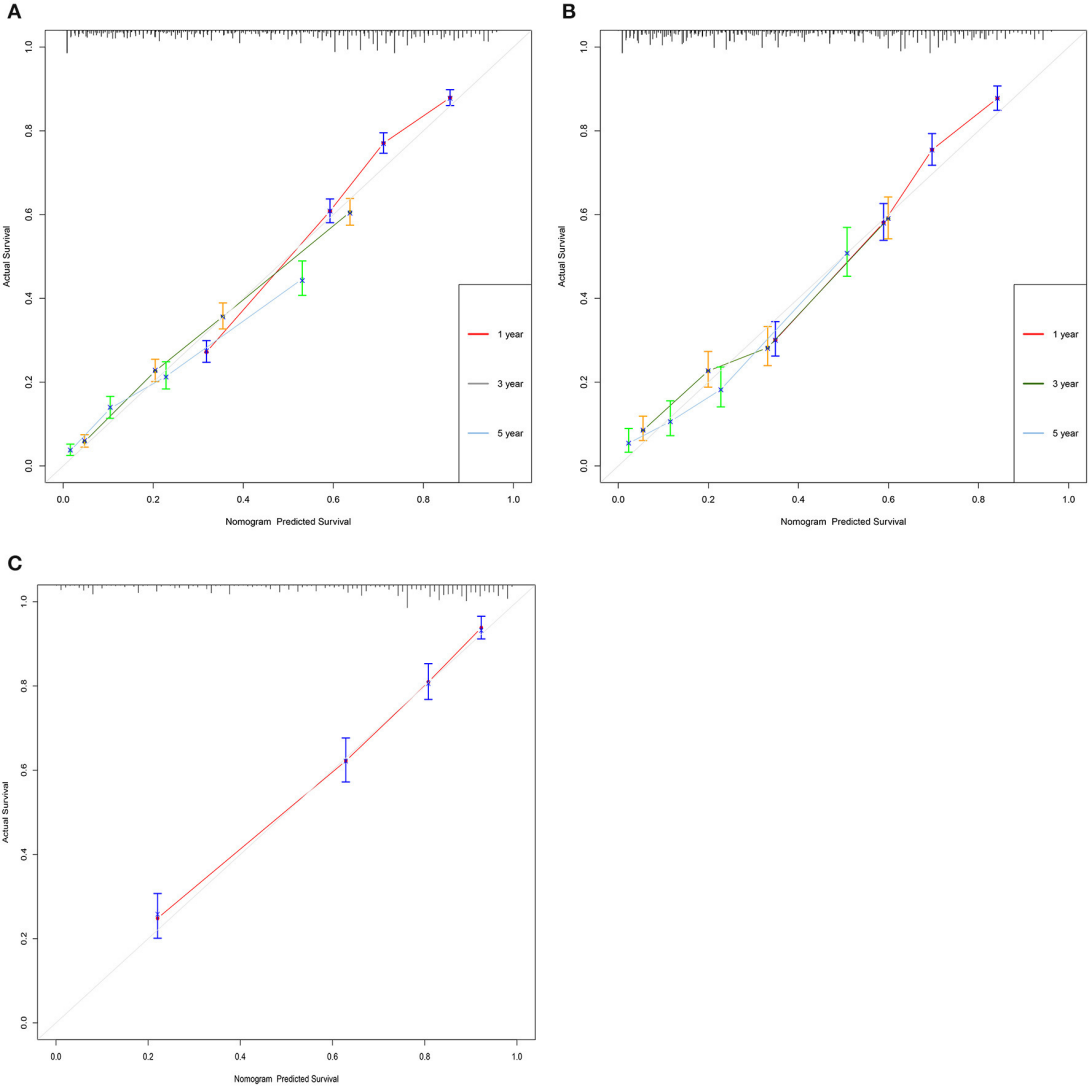
Calibration curves of the nomogram. **(A)** For 1-, 3-, and 5-year OS in training cohort; **(B)** for 1-, 3-, and 5-year OS in validation cohort; and **(C)** for 1-, 3-, and 5-year OS in validation cohort.

**Figure 4:**
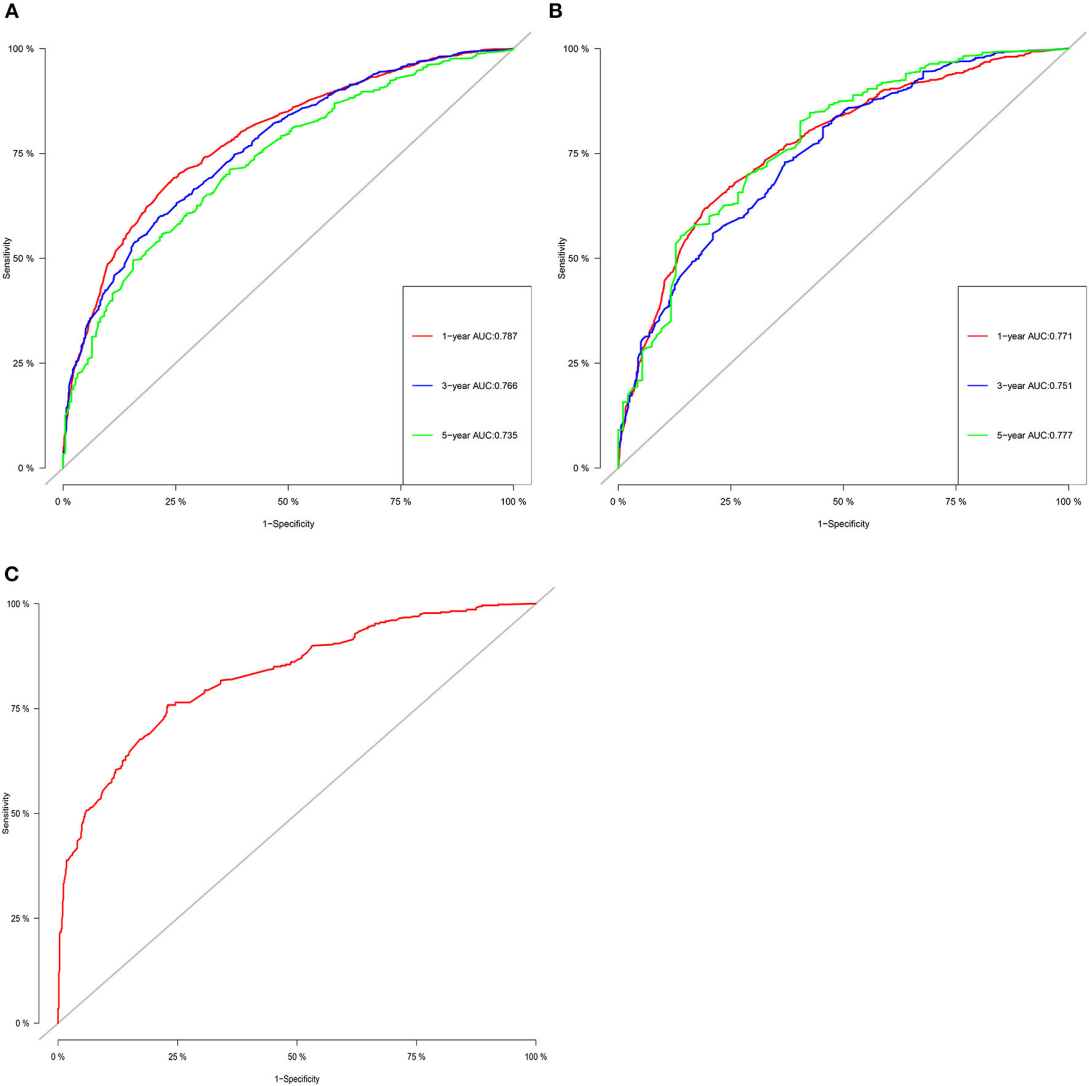
The ROC for OS of 1-, 3- and 5-year of training cohort **(A)**, validation cohort **(B)**, and external validation cohort **(C)**.

The C-index of the training set and the validation set were 0.725 (95% CI: 0.715–0.735) and 0.711 (95% CI: 0.695–0.727), respectively, indicating that the nomogram has good discriminatory power. In the external validation cohort, C-index was 0.797 (95% CI: 0.778–0.816). The calibration plots for the training cohort and the validation cohort used to predict OS show an excellent consistency between the actually observed and nomogram-predicted survival (Figures 3A–C). In the training cohort, the AUC of the predicted nomogram for 1-, 3-, and 5-year were 0.787, 0.766, and 0.35 (Figure 4A). In the validation cohort, the AUC of the predicted nomogram for 1-, 3-, and 5-year were 0.771, 0.751, and 0.777 (Figure 4B), respectively. Furthermore, most patients do not survive longer than 35 months. In the external validation, the AUC of the predicted nomogram for 1 year was 0.828 (Figure 4C).

### Clinical Application of the Nomogram

**Figure 5:**
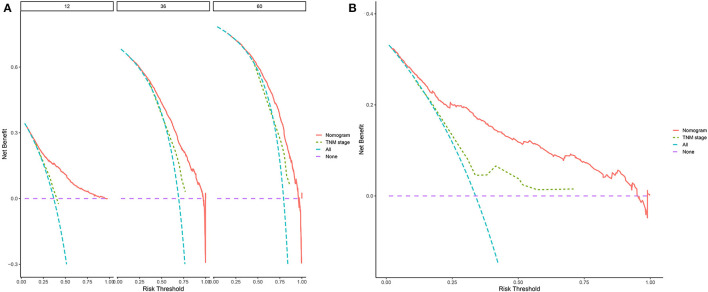
Decision curves of the nomogram predicting OS in validation cohort **(A)** and external validation cohort **(B)**. The y-axis represents the net benefit, and the x-axis represents the threshold probability. The purple line indicates that no patients have died, and the blue line indicates that all patients have died. When the threshold probability is between 20 and 60%, the net benefit of the model exceeds all deaths or no deaths.

**Figure 6:**
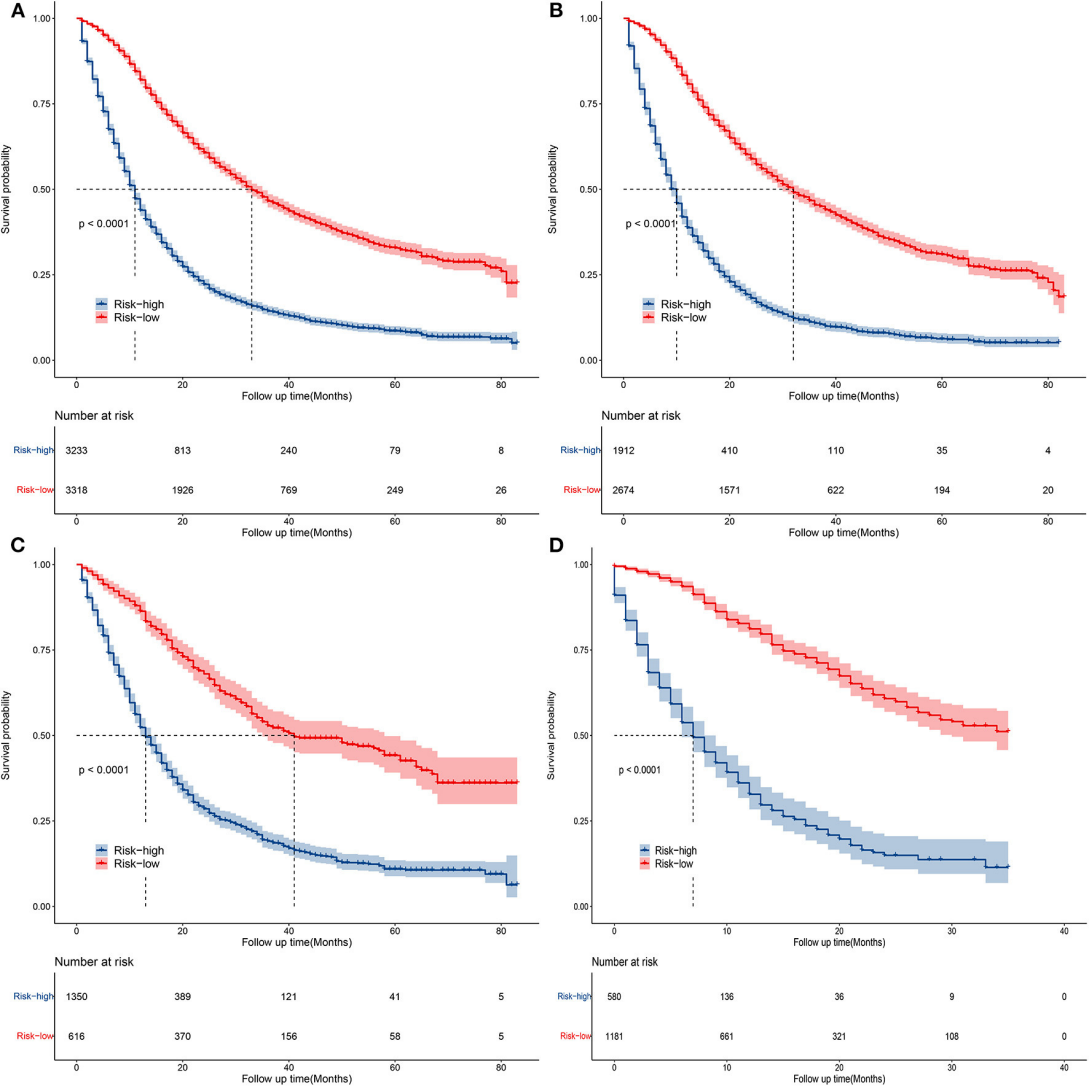
Kaplan–Meier curves of OS for patients in the low- and high-risk groups in all cohorts **(A)**, training cohort **(B)**, validation cohort **(C)**, and external validation cohort **(D)**.

Decision curve analyses (DCAs) showed that the clinical application value of nomograms was superior to that of the traditional TNM stage system (Figures 5A,B). In addition, we constructed a risk stratification system based on the total points of patients on a nomogram. All patients were divided into a risk-low group (overall score ≤ 185.1) and a risk-high group (total score >185.1). The Kaplan–Meier curves of the validation and training sets demonstrated that there were significant differences in the survival of patients in each risk group (Figure 6). That is to say, Kaplan–Meier curve results well-proved the discriminant ability of the nomogram prediction model.

### Online Application for OS Prediction

Based on this nomogram, we have developed a network calculator to predict the OS of elderly patients with PC, which can be accessed at https://yixianainomogram.shinyapps.io/DynNomapp/. Enter the clinicopathological characteristics of the patient, you can immediately get the predictive survival probability of the patient. In conclusion, this online prediction tool is simple, easy to understand, and easy to use in clinical practice.

## Discussion

In this study, we used abundant clinical samples to establish a nomogram to effectively predict 1-, 3-, and 5-year OS in elderly patients with PC based on the SEER database. At the same time, we build the nomogram model and validate the performance of the model by screening clinically significant variables. We determined that surgery, grade, radiation, T stage, N stage, AJCC stage, chemotherapy, age, and insurance were independent factors affecting the OS of elderly patients with PC by univariable and multivariable regression analysis based on the SEER database. Furthermore, a combination of key elements of traditional staging systems and other tumor-associated indicators, such as age, grade, surgery, and chemotherapy, ensures that the nomograms display excellent discriminant ability in predicting OS compared with 8th edition TNM staging systems, which can be seen from the comparison of c-index and values of AUC (19, 20).

Nomogram, a simple statistical predictive tool, has been constructed in PC previously and proved to be useful and effective. Several nomograms have been constructed in patients with PC, and show more accurate survival prediction than the conventional staging system in different populations (8, 13, 14). Although many studies have confirmed that the clinical characteristics and prognosis of older patients with PC are different from young patients (9–11), the nomogram of PC in previous studies cannot accurately predict the OS of elderly patients with PC (12–14). Compared with patients with PC of younger ages, elderly patients with PC have unique physiological characteristics. These characteristics include more comorbidities, less life expectancy, and worse life expectancy. Therefore, it was clear that there was an urgent need to develop predictive models for elderly patients with PC. Previous studies have found that age was an independent factor affecting OS in patients with PC (12–14, 21). Shi et al. (8) have found that the nomograms could effectively predict OS and CSS in young patients with PC, which helps clinicians more accurately and quantitatively judge the prognosis of individual patients. Li and Liu (13) have found that their nomogram for OS predicting can serve as an efficacious survival-predicting model and assist in accurate decision-making for patients over 40 years old with surgically resected PC. The clinicopathological features and prognosis of young patients with PC are not quite the same as those of elderly patients with PC (9–11, 22, 23). Another study used several variables of age, differentiation, TNM stage, surgery, and lymph node surgery to construct a nomogram predicting OS rates in patients with PC having age groups of 25–39, 40–59, 60–79, and 80+ (8). But the study still had some restrictions, they did not include patients who have undergone chemotherapy or radiotherapy. To our knowledge, previous studies found that radiotherapy and chemotherapy can effectively improve the prognosis of post-operative patients with PC (24, 25). Our study found that among elderly patients with PC, those who had received chemotherapy and radiotherapy accounted for ~55.69% and 23.03% of the total number of patients, respectively, which was consistent with the previous study. Therefore, it is necessary to establish and validate a reliable prognostic model for elderly patients with PC with better applicability.

Survival was estimated by the Kaplan–Meier method, and any differences in survival were evaluated with a stratified log-rank test. Multivariable analyses with the Cox proportional-hazards model were used to estimate the simultaneous effects of prognostic factors on survival. Interactions with prognostic factors were also examined with the Cox proportional-hazards model. Kaplan–Meier curves and log-rank tests study the relationship between a single variable and survival, and Kaplan–Meier and log-rank tests are only applicable to categorical variables, but not to numerical variables. The advantage of Cox proportional hazards regression analysis is that it can analyze categorical and numerical variables, and expand the scope of survival analysis from univariate to multivariate analysis (26). By univariate and multivariate Cox proportional hazard regression, several independent prognostic determinants were found to significantly affect OS, namely, age, T stage, histological grade, surgery, radiotherapy, chemotherapy, insurance, and stage of AJCC. We used these factors to construct and validate such prognostic nomograms to predict the probability of OS, and it is gratifying that the model manifested good discrimination and calibration, which meant it might act as a quantitative model to assess the probability of OS in patients with PC. At present, surgery is usually considered the optimal curative option for PC. It would always be recommended if the tumors were resectable. In our study, surgery had the greatest influence on OS in elderly patients with PC, followed by grade, AJCC stage, and T stage. As shown in this study, in most patients, resection of the masses was still the first option to achieve a longer OS with a significant *p*-value < 0.001, indicating that extended pancreatoduodenectomy or local excision of the tumor should be considered even if the procedure is hard to conduct (27). Overall, surgical treatment has better OS than non-surgical treatment. The variance of tumor location in the pancreas can often be tackled in different ways, which determines the prognosis of patients. There still needs to be a larger randomized clinical research to further confirm it. Thus, the therapy selection of surgery decision was another robust indicator for prognosis in terms of the C-index.

The majority of patients with pancreatic adenocarcinoma are over 60 years of age (28, 29). In addition, it has been indicated that increasing age appears to be an important prognostic determinant that directly affects tumor-specific mortality. Further stratified survival analysis showed that patients older than 74 years had lower survival rates compared with those ages fell between 65 and 74 years old. This result resembled other studies which suggested that increasing age might helpful for the mortality of patients (14, 21). Furthermore, the previous study found that three-fourths of non-cancer-specific mortality was observed in elderly patients (13). Age-related comorbid conditions or complications may contribute to non-cancer-specific mortality in elderly patients with PC. This may be related to the decline in the function of the immune system caused by the aging of the patient, which in turn leads to the deterioration of the tumor and shortens the survival time of the patient. Therefore, it is of great significance to take non-cancer-specific mortality into account in the analysis of prognosis, especially in elderly patients.

As we knew, the TNM staging system or differentiation grade is a conventional method for doctors and researchers to evaluate cancer prognosis and select appropriate treatment strategies with respect to the survival of individual patients was still imprecise owing to it being relatively heterogeneous (30, 31). Therefore, the prognostic prediction model seems to be particularly valuable, especially for those who suffer from PC disease. In recent years, studies about the prognosis of PC had been reported after the patients received surgical resection, chemotherapy, or radiotherapy (32–35). Tumor differentiation and TNM stage were commonly regarded as important factors in many cancers and some recent reports even combined these factors with other biomarkers to improve the prediction power (36–40). The performance of the nomogram was measured by the C-index and the area under the receiver operating characteristic (ROC) curve (AUC). All nomogram C-indices were >0.7, the larger the C-index, the more precise was the prognostic prediction, indicating that the model had good discriminatory power. AUC was used as a performance indicator to measure the merit of a machine learning model or a predictive model, a larger AUC (range 0.5–1.0) reflected a more accurate prediction. As indicated in this study, the ROC curves demonstrated that the nomogram showed better discrimination and the ability to provide an individualized prediction for patients. We proposed a nomogram that contains easily measurable clinical features, it is superior to the existing TNM staging system and is more conducive to patient counseling and the promotion of personalized treatment. Univariate analysis and logistic regression were performed to evaluate the impact of clinicopathologic and treatment variables on survival and find potential risk factors. Patients with advanced T stage and AJCC stage suffered from higher mortality and poorer survival rate as demonstrated by multivariate Cox regression analysis. Consistent with previous studies, carcinoma grade is a significant prognostic risk indicator (41). The nomogram model suggests that showed worse clinical outcomes when carcinoma grade shifted to poor differentiation from good differentiation.

Previous studies revealed that female, black, and unmarried patients had a worse OS (8), which was inconsistent with our study. Our study found that no significant difference existed in OS between patients with different marital statuses, gender, and race. Moreover, the multivariable analysis found that insurance has a significant impact on the OS of patients with PC. It is not difficult to imagine that medical insurance will directly affect the patient's choice of treatment, which will contribute to directly affecting the OS. Decision curve analysis (DCA) is a new calculation method that estimates the net benefits under various risk thresholds to evaluate the clinical value of the model. DCA was used to assess the clinical practicability of the model and compare it with T staging, which was a method for evaluating alternative diagnostic or prognostic tools that had advantages over others (42, 43). In this study, we used DCA to validate the accuracy and predictive ability of nomograms for elderly patients with PC, good clinical utility was indicated in the proper range. Validation of the nomogram was significant to avoid overfitting the model and determining representativeness (44). Calibration plots were used to assess the predictive accuracy of the model. In this study, the calibration curve described revealed excellent predictive performance, DCA showed superior net benefits than those of the TNM staging system, which could effectively guarantee the reliability of the constructed nomogram. Moreover, Kaplan–Meier curves showed that the OS of patients in the risk-low and risk-high groups was significantly different from each other, indicating that our model provides accurate surgical intervention and monitoring for high-risk groups, which implied good clinical application potential. Furthermore, we used data from 1,761 patients between 2016 and 2018 as external validation. The survival prediction showed that our prediction model has good accuracy and reliability. They formulated an online application for OS prediction with common and easy-accessible factors, which is convenient for clinical use.

Nevertheless, some limitations to our study should be considered as follows and also some remaining issues for the future. First, this is just a retrospective study. Next, although both the C-index and the calibration curve performed well, validation using a large other external data is still required to further evaluate the predictive accuracy and reliability of our models. Finally, other potential PC-related prognostic factors, such as the family history of PC, obesity, smoking, chronic pancreatitis, history of diabetes, pre-operative nutritional support, and vascular invasion, were not available in the SEER database.

To resolve the above-mentioned limitations, in the future, we aim at implementing the proposed model to determine its real-time applicability and scalability, and we will take other potential PC-related prognostic factors into account during the construction and validation of the model, such as the family history of PC, obesity, smoking, chronic pancreatitis, history of diabetes, and pre-operative nutritional support, lymph node metastasis, and vascular invasion. Besides, methods driven by artificial intelligence (AI) such as deep learning and reinforcement learning will be considered in the formulation of the predictive model, which can help predict specific parameters, hazards, and outcomes (45, 46). Because the blockchain is employed to ensure trust among entities, data immutability, availability, and information security, a predictive model that combines AI and blockchain technology to analyze and integrate clinical characteristics of patients will also be further studied (47, 48). AI and ML could make predictive models smarter and more interesting. Different ML concepts could be added to predictive models to induce a dynamic prediction model. Furthermore, the feasibility of the models could also be studied by examining the computational complexity (49).

## Conclusions

In conclusion, we analyzed the clinicopathological factors determining OS of elderly patients with PC using a large population-based SEER database. Cancer-specific mortality and competing risk mortality were evaluated. Furthermore, nomograms for predicting 1-, 2- and 3-year OS in elderly patients with PC were established for the first time based on a large study cohort. This constructed nomogram showed good performance and can help doctors and elderly patients with PC to more accurately and conveniently predict individual survival and formulate treatment and follow-up strategies.

## Data Availability Statement

Publicly available datasets were analyzed in this study. This data can be found here: https://seer.Cancer.gov/.

## Ethics Statement

The data of this study are obtained from the SEER database. The data of patients are public and anonymous, so this study does not require ethical approval and informed consent.

## Author Contributions

JZ, XinL, YZ, XiaL, and SP contributed to the conception and design. XiaL, JZ, YZ, and SP collected and analyzed the data. SP and YZ drew the figures and tables. SP and XinL wrote the draft. JC, YL, CL, JQ, and XG contributed to manuscript writing and revision. All authors approved the final manuscript.

## Conflict of Interest

The authors declare that the research was conducted in the absence of any commercial or financial relationships that could be construed as a potential conflict of interest.

## Publisher's Note

All claims expressed in this article are solely those of the authors and do not necessarily represent those of their affiliated organizations, or those of the publisher, the editors and the reviewers. Any product that may be evaluated in this article, or claim that may be made by its manufacturer, is not guaranteed or endorsed by the publisher.
