# Supplementary material for: A Visualized Dynamic Prediction Model for Overall Survival in Elderly Patients With Pancreatic Cancer for Smart Medical Services
Source: Front Public Health. 2022 May 24;10:885624. doi: 10.3389/fpubh.2022.885624 (PMC9171143; doi:10.3389/fpubh.2022.885624)
Supplement: Supplementary file 1 [file Table_1.docx]

Table S1.Demographical and Clinical characteristics of external validation set.

| Characteristic | All patients |
| --- | --- |
|  | N=1761 |
| Age (%) |  |
| 65-74 | 1019 (57.86) |
| 74+ | 742 (42.14) |
| Sex (%) |  |
| Female | 863 (49.01) |
| Male | 898 (50.99) |
| Race (%) |  |
| White | 1423 (80.81) |
| Black | 146 (8.29) |
| Other | 192 (10.90) |
| Marital.status (%) |  |
| No | 675 (38.33) |
| Married | 1086 (61.67) |
| Grade (%) |  |
| I | 258 (22.07) |
| II | 559 (47.82) |
| III | 342 (29.26) |
| IV | 10 (0.86) |
| T (%) |  |
| T1 | 249 (15.97) |
| T2 | 526 (33.74) |
| T3 | 784 (50.29) |
| N (%) |  |
| N0 | 940 (59.61) |
| N1 | 637 (40.39) |
| AJCC (%) |  |
| IA | 205 (11.64) |
| IB | 308 (17.49) |
| IIA | 590 (33.50) |
| IIB | 658 (37.37) |
| Surg (%) |  |
| No | 542 (30.78) |
| Extended pancreatoduodenectomy | 28 (1.59) |
| Local excision of tumor | 322 (18.29) |
| Local or pancreatectomy | 777 (44.12) |
| Total pancreatectomy | 92 (5.22) |
| Radiation (%) |  |
| No | 1458 (82.79) |
| Yes | 303 (17.21) |
| Chemotherapy (%) |  |
| No | 712 (40.43) |
| Yes | 1049 (59.57) |
| Tumor.Size (median [IQR]) | 32.000 [23.000, 42.000] |
